# Novel lineage of anelloviruses with large genomes identified in dolphins

**DOI:** 10.1128/jvi.01370-24

**Published:** 2024-12-12

**Authors:** Matthew D. De Koch, Mart Krupovic, Russell Fielding, Kendal Smith, Kelsie Schiavone, Katharine R. Hall, Vincent S. Reid, Diallo Boyea, Emma L. Smith, Kara Schmidlin, Rafaela S. Fontenele, Eugene V. Koonin, Darren P. Martin, Simona Kraberger, Arvind Varsani

**Affiliations:** 1 The Biodesign Center for Fundamental and Applied Microbiomics, Center for Evolution and Medicine, School of Life Sciences, Arizona State University 7864 , Tempe, Arizona, USA; 2 Institut Pasteur, Université Paris Cité, CNRS UMR6047, Archaeal Virology Unit 555089 , Paris, France; 3 HTC Honors College, Coastal Carolina University 2672 , Conway, South Carolina, USA; 4 Department of Earth and Environmental Systems, The University of the South 7303 , Sewanee, Tennessee, USA; 5 Barrouallie Whaler’s Project, Barrouallie, Saint Vincent and the Grenadines; 6 Independent Researcher, Barrouallie, Saint Vincent and the Grenadines; 7 Department of Chemical and Biological Sciences, The University of the West Indies at Cave Hill 41633 , Bridgetown, Saint Michael, Barbados; 8 National Center for Biotechnology Information, National Library of Medicine 42710 , Bethesda, Maryland, USA; 9 Computational Biology Division, Department of Integrative Biomedical Sciences, Institute of Infectious Diseases and Molecular Medicine, University of Cape Town 517108 , Observatory, Western Cape, South Africa; 10 Structural Biology Research Unit, Department of Integrative Biomedical Sciences, University of Cape Town 517108 , Rondebosch, Cape Town, South Africa; College of Agriculture & Life Sciences, University of Arizona, Tucson, Arizona, USA

**Keywords:** pantropical spotted dolphin, killer whale, short-finned pilot whale, false killer whale, Delphinidae, *Anelloviridae*, *Qoptorquevirus*, single jelly-roll fold

## Abstract

Anellovirus infections are ubiquitous in mammals but lack any clear disease association, suggesting a commensal virus-host relationship. Although anelloviruses have been identified in numerous mammalian hosts, their presence in members of the family Delphinidae has yet to be reported. Here, using a metagenomic approach, we characterize complete anellovirus genomes (*n* = 69) from four Delphinidae host species: short-finned pilot whale (*Globicephala macrorhynchus*, *n* = 19), killer whale (*Orcinus orca*, *n* = 9), false killer whale (*Pseudorca crassidens*, *n* = 6), and pantropical spotted dolphin (*Steno attenuatus*, *n* = 1). Sequence comparison of the open reading frame 1 (ORF1) encoding the capsid protein, the only conserved gene shared by all anelloviruses, shows that the Delphinidae anelloviruses form a novel genus-level clade that encompasses 22 unique species-level groupings. We provide evidence that different Delphinidae species can be co-infected by multiple anelloviruses belonging to distinct species groupings. Notably, the ORF1 protein of the Delphinidae anelloviruses is considerably larger than those encoded by all previously described anelloviruses from other hosts (spanning 14 vertebrate orders and including 27 families). Comprehensive analysis of the ORF1 sequences and predicted protein structures showed that the increased size of these proteins results from divergent elaborations within the capsid-distal P2 subdomain and elongation of the C-terminal domain of ORF1. Comparative structural and phylogenetic analyses suggest that acquisition of the P2 subdomain and its diversification occurred convergently in the anelloviruses associated with primate and Delphinidae hosts. Collectively, our results further the appreciation of diversity and evolution of the ubiquitous and enigmatic viruses in the family *Anelloviridae*.

**IMPORTANCE:**

Anelloviruses are ubiquitous in mammals, but their infection has not yet been linked to any disease, suggesting a commensal virus-host relationship. Here, we describe the first anelloviruses associated with diverse species of dolphins. The dolphinid anelloviruses represent a new genus (tentatively named “Qoptorquevirus”) and encode open reading frame 1 (ORF1) (capsid) proteins that are considerably larger than those encoded by previously described anelloviruses from other hosts. Comprehensive analysis of the ORF1 sequences and predicted protein structures revealed the underlying structural basis for such an extravagant ORF1 size and suggested that ORF1 size increased convergently in the anelloviruses associated with primate and Delphinidae hosts, respectively. Collectively, our results provide insights into the diversity and evolution of *Anelloviridae*. Further exploration of the anellovirus diversity, especially in the host species that have not yet been sampled, is expected to further clarify their evolutionary trajectory and explain the unusual virus-host commensal relationship.

## INTRODUCTION

Anelloviruses have small icosahedral virions that encapsidate negative-sense, single-stranded circular DNA genomes. These viruses have been identified in a variety of terrestrial vertebrate hosts, yet their prevalence and diversity in marine mammals remain poorly understood (1). Anelloviruses are classified into the family *Anelloviridae*, which currently includes ~30 genera comprising ~150 species, although due to the extensive diversity of anelloviruses, their taxonomy is in constant flux. The vast majority of the known anellovirus hosts are mammals, with members of only one genus infecting birds (1, 2). Mammalian anellovirus genomes range in size from 1.6 to 3.9 kb and contain one large and two or three smaller protein-coding open reading frames (ORFs). The exact functions of these genes are not entirely known, especially those of the smaller ORFs. ORF1 is the signature ORF of anelloviruses and encodes a single jelly-roll (JR) capsid protein with an N-terminal arginine-rich domain, similar to the DNA-binding R-arm found in the capsid proteins of circoviruses and many other viruses with icosahedral capsids and ssRNA or ssDNA genomes (3). The smaller ORF2 contains a serine-rich region, which likely binds template DNA (4). However, the molecular mechanisms of anellovirus replication remain enigmatic because their small genomes encode no proteins homologous to any known replication enzymes (3), and neither helper viruses nor the involvement of the host replication machinery has been demonstrated.

Following the initial discovery of an anellovirus (named torque teno virus) in the blood of a post-transfusion patient from Japan in 1997 (5), related viruses have been found in various animals including non-human primates (6–8), pinnipeds (9–11), birds (12), felids (13, 14), bears (3, 15), and rodents (16, 17). Transmission of anelloviruses is believed to mainly occur via the fecal-oral and saliva routes (18).

The key characteristic of anelloviruses, which continues to puzzle researchers, is their ubiquity in vertebrates without a clear disease association. Anellovirus prevalence has been shown to range from 5% to 90% in human populations, with initial presence often detected in the first few months following birth (4). Anellovirus DNA has been detected in whole blood, saliva, tissues, and feces (19–23). Although a few studies have suggested possible associations with certain viral infections (24) and disease (25–27), detailed investigation of these potential relationships has been hindered by the omnipresence of anelloviruses and the lack of robust cell culture models (28). One popular hypothesis is that anelloviruses are commensals or even mutualists of their hosts, being a part of the so-called healthy human virome (4, 29).

Although anelloviruses have been observed in numerous terrestrial mammalian species, very few studies have investigated their prevalence and diversity in marine mammals. Currently, pinnipeds remain the only marine mammals that have been assessed for the presence of anelloviruses (3, 9–11, 30). To close this knowledge gap, we accessed archived samples collected for other studies of Delphinidae species in the Caribbean. In particular, we analyzed the presence of anelloviruses in tissue samples from four species: short-finned pilot whale (*Globicephala macrorhynchus*), killer whale (*Orcinus orca*), false killer whale (*Pseudorca crassidens*), and pantropical spotted dolphin (*Stenella attenuata*). Although Delphinidae population demographics are often difficult to accurately estimate, historic whaling data indicate that the four species studied here are among the most abundant cetacean species in St. Vincent and the Grenadines (31). We have previously identified a polyomavirus and cressdnaviricots in killer whale and short-finned pilot whale samples (32). In this study, we build on this previous work with a focus on anelloviruses. We recovered 69 anellovirus genomes that fall into 22 species groupings (TTDelV g1 through g22) within a putative new genus of Delphinidae-infecting anelloviruses. Comprehensive phylogenetic analyses coupled with comparative structural analysis of the ORF1 proteins provided insights into the diversity and evolution of these viruses.

## MATERIALS AND METHODS

### Sample collection

Tissue samples from four Delphinidae species (short-finned pilot whale, killer whale, false killer whale, and pantropical spotted dolphin) were obtained through collaboration with artisanal subsistence whalers from the Caribbean island of St. Vincent, where local communities have carried out this traditional practice for more than a century (33). The various tissue samples (kidney, liver, or muscle) were collected from the deceased animals with the permission of the whalers and associated partners.

The tissue samples were first imported to the University of the South (Sewanee, TN, USA), and a subsample of each sample was transported to Arizona State University (Tempe, AZ, USA). These subsamples were stored at −80°C until processing.

### Viral nucleic acid isolation and high-throughput sequencing

The tissue samples (kidney, liver, or muscle) were collected from 55 marine mammals between 2015 and 2016 (Table 1). From each sample, ~5 g of tissue was individually homogenized in SM buffer (G-Biosciences, USA). Viral DNA was then extracted from 200 µL of each homogenate, using the High Pure Viral Nucleic Acid Kit (Roche Diagnostics, USA), following the manufacturer’s protocol. To enrich circular DNAs, we carried out rolling-circle amplification (RCA), using the phi29 DNA Polymerase Kit (Watchmaker Genomics, USA).

**TABLE 1:** Summary of sample information for all anelloviruses recovered in this study including source/host, Delphinidae host family demographic information, sampling location, sample date, sample type, anellovirus species grouping, and anellovirus genome accession number

Sample ID	Host species	Sampling location	Sample date	Sample type	Virus species grouping	Accession no.
USW2	*Stenella* spp.	St. Vincent island	3-August-15	Kidney	TTDelV g18	PP790224
					TTDelV g14	PP790225
USW5	*Orcinus orca*	St. Vincent island	26-August-15	Muscle, fresh	TTDelV g20	PP790226
					TTDelV g22	PP790227
					TTDelV g22	PP790228
USW7	*Orcinus orca*	St. Vincent island	26-August-15	Liver	TTDelV g21	PP790229
					TTDelV g19	PP790230
					TTDelV g16	PP790231
USW8	*Orcinus orca*	St. Vincent island	26-August-15	Muscle, fresh	TTDelV g20	PP790232
					TTDelV g20	PP790233
USW9	*Globicephala macrorhynchus*	St. Vincent island	22-September-15	Muscle, fresh	TTDelV g8	PP790234
					TTDelV g5	PP790235
					TTDelV g8	PP790236
USW10	*Globicephala macrorhynchus*	St. Vincent island	22-September-15	Kidney	TTDelV g8	PP790237
					TTDelV g8	PP790238
					TTDelV g8	PP790239
USW11	*Globicephala macrorhynchus*	St. Vincent island	28-September-15	Muscle, fresh	TTDelV g8	PP790240
					TTDelV g8	PP790241
USW12	*Pseudorca crassidens*	St. Vincent island	7-October-15	Muscle, fresh	TTDelV g3	PP790242
					TTDelV g3	PP790243
					TTDelV g3	PP790244
USW13	*Pseudorca crassidens*	St. Vincent island	12-October-15	Muscle, fresh	TTDelV g4	PP790245
USW15	*Pseudorca crassidens*	St. Vincent island	29-October-15	Liver	TTDelV g3	PP790246
					TTDelV g3	PP790247
USW16	*Pseudorca crassidens*	St. Vincent island	29-October-15	Kidney	TTDelV g8	PP790248
					TTDelV g3	PP790249
USW17	*Pseudorca crassidens*	St. Vincent island	29-October-15	Muscle, fresh	TTDelV g3	PP790250
					TTDelV g3	PP790251
					TTDelV g3	PP790252
USW18	*Pseudorca crassidens*	St. Vincent island	4-November-15	Liver	TTDelV g6	PP790253
					TTDelV g4	PP790254
					TTDelV g2	PP790255
USW22	*Globicephala macrorhynchus*	St. Vincent island	13-March-16	Muscle, fresh	TTDelV g8	PP790256
USW23	*Globicephala macrorhynchus*	St. Vincent island	13-March-16	Liver	TTDelV g8	PP790257
					TTDelV g8	PP790258
USW24	*Globicephala macrorhynchus*	St. Vincent island	13-March-16	Liver	TTDelV g8	PP790259
					TTDelV g8	PP790260
USW25	*Globicephala macrorhynchus*	St. Vincent island	13-March-16	Kidney	TTDelV g1	PP790261
					TTDelV g1	PP790262
USW26	*Globicephala macrorhynchus*	St. Vincent island	13-March-16	Muscle, fresh	TTDelV g5	PP790263
					TTDelV g5	PP790264
USW28	*Globicephala macrorhynchus*	St. Vincent island	15-March-16	Muscle, fresh	TTDelV g8	PP790265
					TTDelV g13	PP790266
USW29	*Orcinus orca*	St. Vincent island	28-April-16	Muscle, fresh	TTDelV g8	PP790267
USW30	*Orcinus orca*	St. Vincent island	28-April-16	Liver	TTDelV g7	PP790268
					TTDelV g11	PP790269
					TTDelV g2	PP790270
USW35	*Orcinus orca*	St. Vincent island	28-April-16	Liver	TTDelV g17	PP790271
USW36	*Orcinus orca*	St. Vincent island	28-April-16	Muscle, fresh	TTDelV g15	PP790272
USW39	*Globicephala macrorhynchus*	St. Vincent island	27-May-16	Muscle, fresh	TTDelV g2	PP790273
					TTDelV g5	PP790274
					TTDelV g8	PP790275
USW40	*Globicephala macrorhynchus*	St. Vincent island	27-May-16	Muscle, fresh	TTDelV g2	PP790276
USW42	*Orcinus orca*	St. Vincent island	9-June-16	Muscle, fresh	TTDelV g3	PP790277
					TTDelV g3	PP790278
USW44	*Globicephala macrorhynchus*	St. Vincent island	23-June-16	Liver	TTDelV g10	PP790279
					TTDelV g8	PP790280
USW45	*Globicephala macrorhynchus*	St. Vincent island	23-June-16	Liver	TTDelV g1	PP790281
USW48	*Globicephala macrorhynchus*	St. Vincent island	23-June-16	Liver	TTDelV g8	PP790282
					TTDelV g5	PP790283
					TTDelV g8	PP790284
USW49	*Globicephala macrorhynchus*	St. Vincent island	23-June-16	Kidney	TTDelV g1	PP790285
USW50	*Globicephala macrorhynchus*	St. Vincent island	23-June-16	Liver	TTDelV g3	PP790286
					TTDelV g3	PP790287
					TTDelV g12	PP790288
USW51	*Globicephala macrorhynchus*	St. Vincent island	23-June-16	Muscle, fresh	TTDelV g3	PP790289
USW52	*Globicephala macrorhynchus*	St. Vincent island	23-June-16	Kidney	TTDelV g8	PP790289
USW54	*Globicephala macrorhynchus*	St. Vincent island	23-June-16	Muscle, fresh	TTDelV g9	PP790291
USW55	*Orcinus orca*	St. Vincent island	9-June-16	Muscle, fresh	TTDelV g5	PP790292

The RCA products were pooled per animal species, and these were then used to generate libraries using the TruSeq DNA Nano kit (Illumina, Inc.) and sequenced (2 × 100 bp) on an Illumina HiSeq4000 sequencer at Macrogen Inc. (USA). The raw reads were quality trimmed using Trimmomatic version 0.39 (34) and then *de novo* assembled with MEGAHIT version 1.2.9 (35). *De novo*-assembled contigs > 750 nts were screened against a local NCBI viral protein RefSeq database (Release 220) using BLASTx (36).

Based on the *de novo*-assembled anellovirus contigs, we designed a pair of abutting primers to screen each sample and recover complete genomes by PCR. In each reaction, one forward primer (cetacean anellovirus F: 5′-TGA GTT TAC TGC GCS AGY GGT CAA T-3′) was paired with one of two reverse primers (cetacean anellovirus R: 5′-GCC ATT CGT CAC CCC ACT TAC TTA T-3′ or 5′-GCC ATT CGT CAG TGT AGT TAC TTA T-3′). PCR cycling conditions were applied according to primer annealing temperatures of 55°C and the manufacturer’s recommendations. The amplicons were resolved on a 0.7% agarose gel, and the amplicons of ~3.5–3.8 kb were excised and purified using the MEGAquick-spin Plus Total Fragment DNA Purification Kit (iNtRON Biotechnology, South Korea). Then, the purified amplicons from each PCR were ligated into the pJET 1.2 vector (Thermo Fisher Scientific, USA) and transformed into XL blue *Escherichia coli*-competent cells. DNA from the recombinant plasmid was extracted using the DNA-spin Plasmid DNA Purification Kit (iNtRON Biotechnology, South Korea) and sequenced at Plasmidsaurus, USA, using Oxford Nanopore long-read sequencing technology.

### Anellovirus sequence analysis

Anellovirus genomes were annotated with Geneious Prime (Dotmatics, USA). A ORF1 data set was assembled from the genomes identified in this study as well as those of representatives from each species available in GenBank (downloaded on 1st December 2023).

The ORF1 sequences were translated and aligned with MAFFT version 7.113 (37). The alignment was trimmed using TrimAl with a 0.2 gap threshold (38), and the resulting trimmed alignment was used to infer a maximum likelihood phylogenetic tree using PhyML 3 (39) using the best fit substitution model VT + G + I determined using ProtTest 3 (40). Branches with less than 0.8 aLRT branch support were collapsed using TreeGraph2 version 2.14 (41).

The ORF1 amino acid sequence lengths characterized in this study were compared to those of representative species across 14 distinct host orders using Rstudio (42) with ggplot2 (43) and tidyr (44) packages. To visualize relative ORF1 amino acid sequence lengths, these data were compiled to construct a jitter dotplot.

Pairwise identity analyses for all ORF1 nucleotide and amino acid sequences were determined using SDT version 1.2 (45). Clinker (46) was used to illustrate genome organization and show the percentage identity of the encoded protein sequences (ORF1, ORF2, and ORF3) of representative genomes from the 22 TTDelV species groupings.

Recombination events evident within the whole genome alignment were detected, and recombination breakpoint distributions were analyzed with RDP5.57 (47) using default parameters. For optimal recombination detection, sequences were auto-masked. Only events detected with >3 different recombination detection methods implemented in RDP5 with phylogenetic support for recombination and a *P*-value of <0.05 were considered credible.

The number of arginine (R) and lysine (K) were counted in the N terminus of the ORF1 protein of all the representative anelloviruses and used to determine a Pearson correlation coefficient with *orf1* gene and genome size using DATAtab (48).

### Protein structure prediction and analysis

ORF1 structures of anelloviruses representing all 22 species described in this study were modeled using AlphaFold2 (49) via a local installation of ColabFold version 1.5.5 (50). Multiple sequence alignments of ORF1 from anelloviruses of delphinids and other hosts were used as an input for modeling, with six recycles. When appropriate, the models were further refined using DeepFold (51) with zero recycles. The quality of the generated structural models was assessed using the local distance difference test (52). The obtained ORF1 structural models of TTDelVs were compared using DALI (53) to the previously published ORF1 models representing all established genera of the *Anelloviridae* family (3). For a more thorough comparison, the structures were aligned using the MatchMaker algorithm (54). Protein structures and structural models were visualized using ChimeraX version 1.7.1 (55).

## RESULTS AND DISCUSSION

### Characterization of anelloviruses from four delphinid species

In this study, a total of 69 complete anellovirus genomes (size range of 3,480–3,780 nts) were sequenced from 35 individuals from four Delphinidae species. A total of 36 anellovirus genomes were recovered from the short-finned pilot whales (*n* = 19), 17 genomes from the killer whales (*n* = 9), 14 genomes from the false killer whales (*n* = 6), and 2 genomes from the pantropical spotted dolphin (*n* = 1). On average, about two distinct anelloviruses were recovered from each sample (Table 1).

Anelloviruses were identified from three tissue types. In total, 11 anellovirus genomes were recovered from kidney samples (*n* = 6); 25 genomes from liver samples (*n* = 11); and 33 genomes from muscle samples (*n* = 18) (Table 1). We did not find any clear correlation between organ types and anelloviruses identified. Although the majority of anelloviruses were recovered from the muscle samples, these samples represented ~50% of our sample types.

Open reading frames ORF1, ORF2, and ORF3 were identified and annotated for all 69 genomes (deposited in GenBank under accession numbers PP790224–PP790292). The killer whale samples had the largest anellovirus genome size range, spanning from 3,480 to 3,780 nts. Anelloviruses from the short-finned pilot whales had a similar range, from 3,505 to 3,766 nts. Those from the false killer whales had a slightly smaller range, from 3,619 to 3,754 nts. Finally, the pantropical spotted dolphin sequences had the smallest genomes, from 3,500 to 3,541 nts. However, this dolphin species (*n* = 1) is the smallest number of recovered anellovirus genomes among the four species. Thus, although in terms of the genome length distribution, TTDelVs fall within the range of previously described anellovirus genomes (1,600–3,900 nts), they clearly include some of the largest genomes described thus far. Notably, all other known anelloviruses that have genomes larger than 3,500 nts infect primates (3).

### Distribution of pairwise genetic distances between delphinid anelloviruses

**Fig 1:**
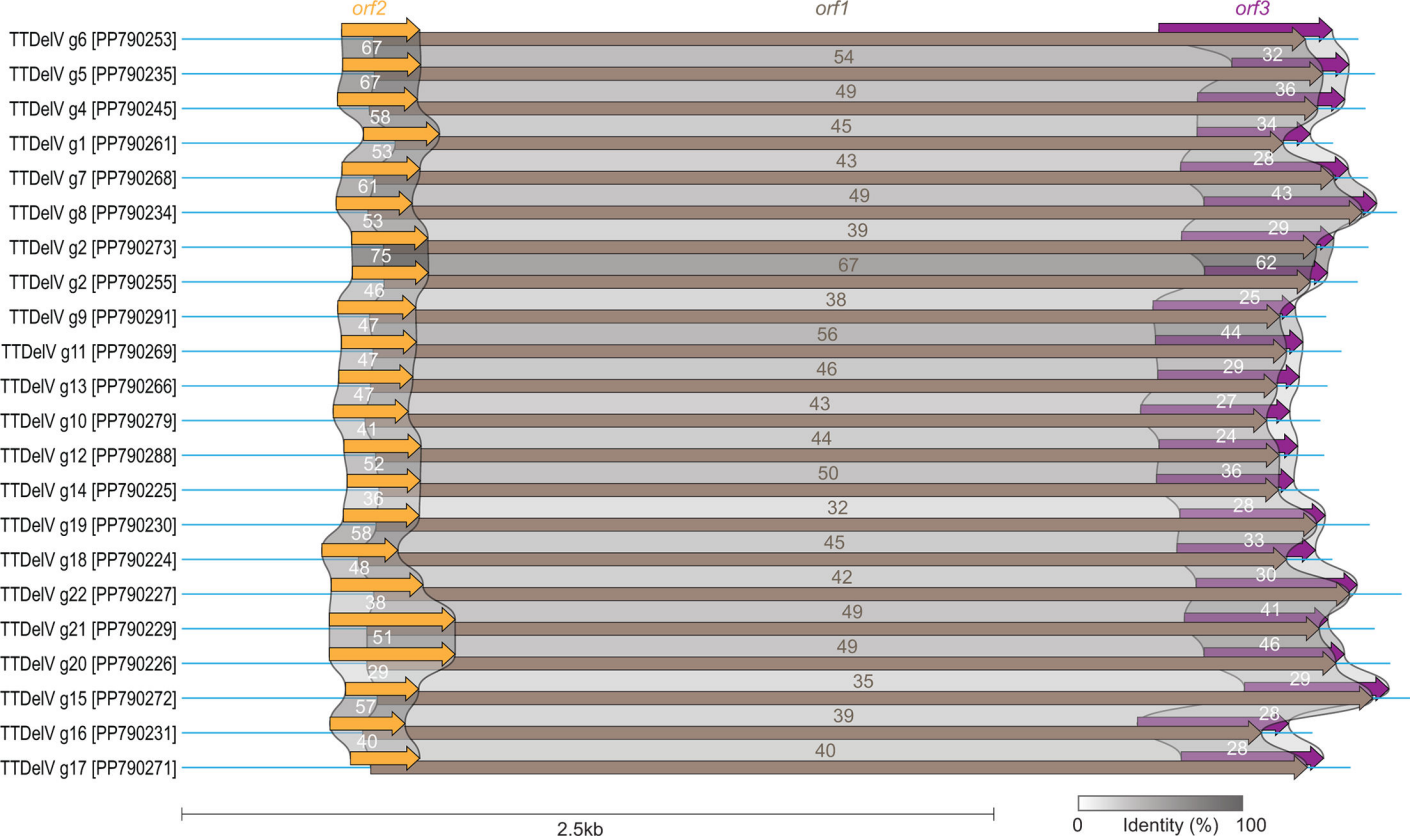
Genome organization of TTDelVs. Genome organization and percentage identity of the encoded protein sequences of ORF1, ORF2, and ORF3 of representative genomes from the 22 TTDelV species groupings.

According to the current ICTV taxonomy guidelines, a 69% ORF1 pairwise nucleotide identity is used as a species demarcation threshold for the classification of viruses in the family *Anelloviridae* (2). Based on this threshold, we can group the 69 anelloviruses from this study into 22 unique species-level assemblages—torque teno Delphinidae virus groups 1 through 22 (TTDelV g1 through g22; Fig. 1; Data S1). Genome organization of the representative genomes from these 22 unique species level assemblages is provided in Fig. 1. The ORF1 nucleotide pairwise identity values range from 55% to 99% similarity among all the 69 TTDelVs (Data S1). At a full genome level, the 69 Delphinidae anelloviruses share 59%–99% identity (Data S1).

The BLASTx analysis revealed that cetacean-infecting anelloviruses are most closely related to porcine-infecting viruses in the genus *Kappatorquevirus*. Given that cetaceans (family Delphinidae) and porcines (family Suidae) both belong to the same mammalian order, Artiodactyla, this observation is compatible with the coevolution of Artiodactyla-infecting anelloviruses with their hosts, following the divergence of the corresponding host lineages from their last common ancestor. Molecular analyses among groups in the Artiodactyla order indicate that the Delphinidae and Suidae families diverged ~50 million years ago (56).

### Anellovirus ORF1 protein phylogeny and species groupings

**Fig 2:**
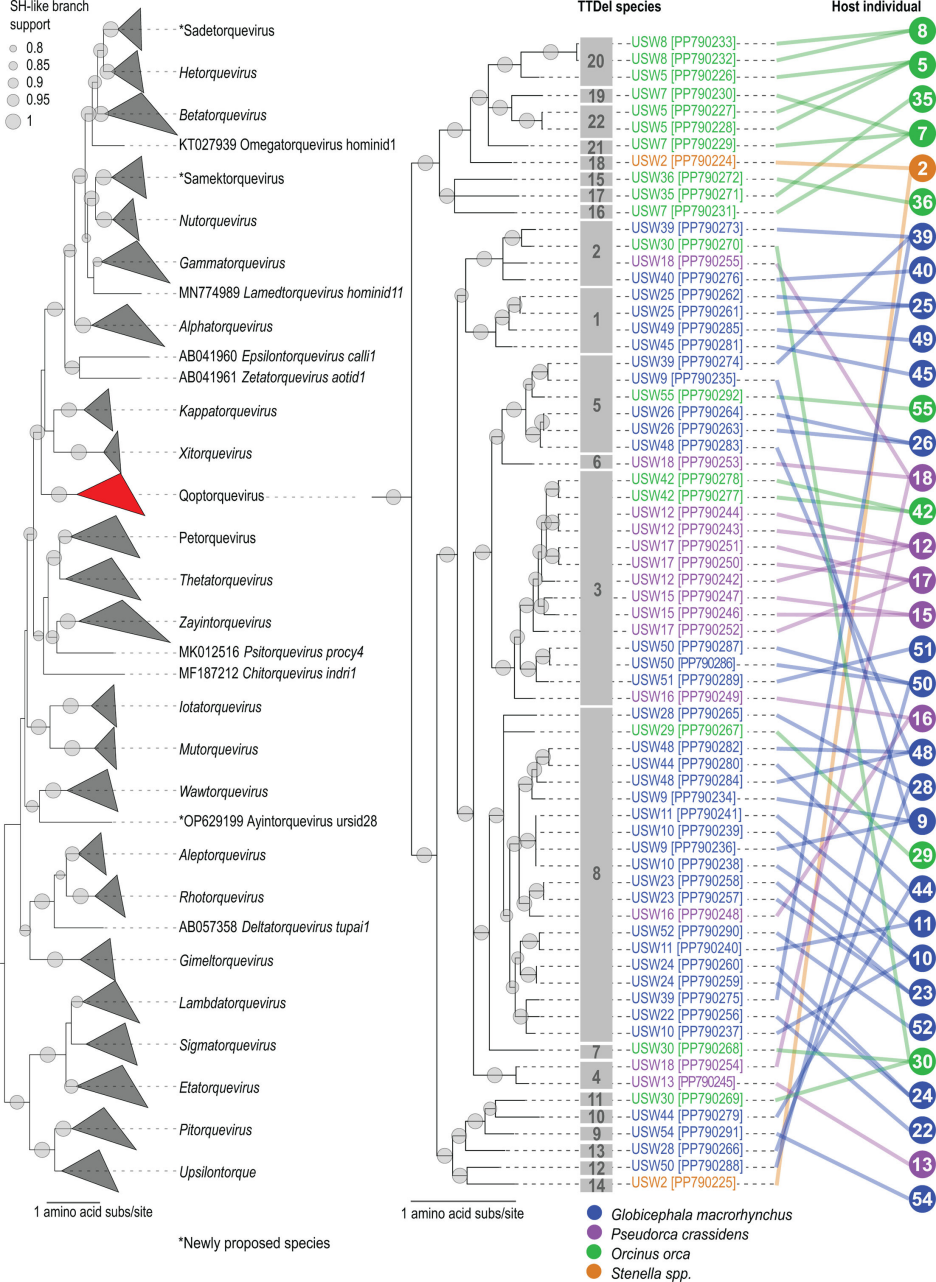
Anelloviruses sampled from Delphinidae species cluster together and reveal diverse co-infections. The maximum-likelihood phylogenetic tree on the left depicts the evolutionary relationships among representative anellovirus species ORF1 proteins together with those recovered from Delphinidae family hosts in this study. All the anellovirus genomes recovered here cluster together, across 22 TTDelV species groupings, in one large clade (shown in red). The clade of the Delphinidae is shown on the right, with a tanglegram for each to show the individual and host species each genome was recovered from. These virus-host relationships reveal co-infection dynamics among the Delphinidae family anelloviruses.

In the phylogenetic tree of the ORF1 amino acid sequences of the anelloviruses characterized in this study and those available from GenBank, the Delphinidae-infecting anelloviruses clustered together in a distinct clade (Fig. 2), representing a putative new genus (proposed genus name “Qoptorquevirus”). The Delphinidae hosts that were considered here all belong to the infraorder Cetacea and the order Artiodactyla. Many of the host tanglegram lines span across several of the 22 TTDelV species groupings. In particular, of the 35 individual cetacean hosts, 23 harbored more than one variant of the anellovirus. Moreover, 11 individual hosts harbored multiple anelloviruses across more than one TTDelV species group. Thus, co-infections with diverse anelloviruses occur across these cetacean hosts, but notably, not beyond the infraorder Cetacea. Individuals co-infected with multiple anelloviruses have been documented broadly in several host groups, including humans (4, 23, 57), pinnipeds (11, 30), ursids (3, 58), rodents (16, 59), and felids (13, 60).

In a few cases, TTDelV species groupings coincide with cetacean host species. For example, 18 of the 20 anellovirus genomes from TTDelV g8 and five of the six anellovirus genomes from TTDelV g5 were identified from short-finned pilot whales. In addition, 9 of the 14 anellovirus genomes from TTDelV g3 were identified in false killer whales. In the majority of the other anellovirus species groupings, however, greater host diversity is observed, suggesting the occasional cross-species transmissions of TTDelVs. At the higher host taxonomic level, anelloviruses appear to have coevolved with their hosts, with no observed spillover to other animals thus far.

### Delphinid anelloviruses display recombination patterns similar to those in other anellovirus groups

**Fig 3:**
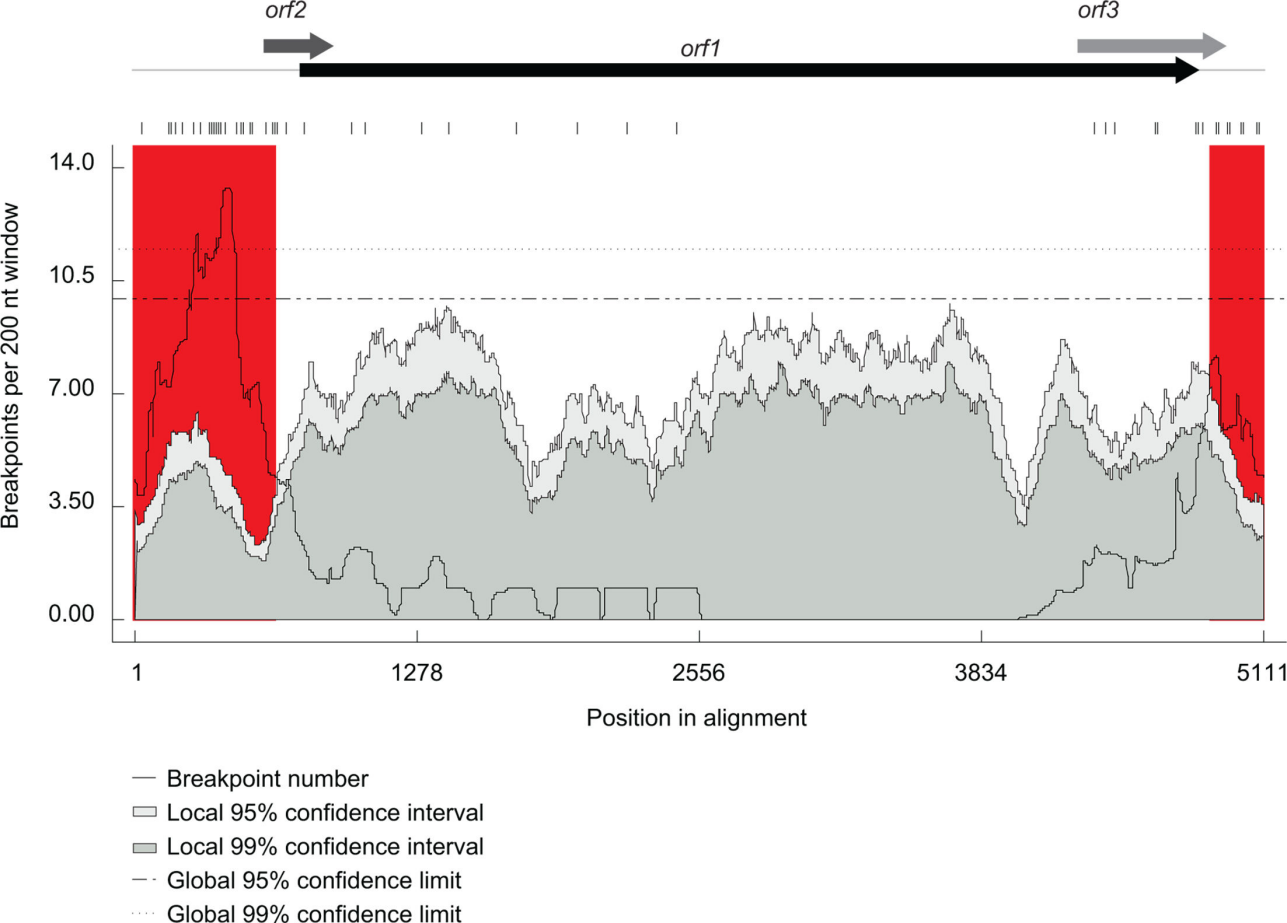
Distribution of recombination breakpoints along delphinid anellovirus genomes. Breakpoint hotspots are indicated in red. The light and dark sage color areas of the plots indicate 95% and 99% confidence intervals, respectively.

The 69 genomes displayed evidence of 30 independent recombination events with detectable recombination breakpoints clustered in the non-coding genome region. This pattern, with recombination breakpoint hotspots in the non-coding region and very few breakpoints detectable in ORF1 (Fig. 3), mirrors that observed in anelloviruses from multiple different genera (3, 11, 13, 23, 61).

In all but 2 of the 30 detected recombination events, one or both of the parental sequences shared <90% sequence identity with the respective genome regions that contributed to recombinants. Furthermore, for 13 of the 30 recombination events, only one parental sequence was identified (Data S2). Together, these observations suggest that the 69 sequences we analyzed comprise a severely under-representative sample of Delphinidae anelloviruses.

### Delphinid anelloviruses encode by far the largest ORF1 proteins within the family *Anelloviridae*

**Fig 4:**
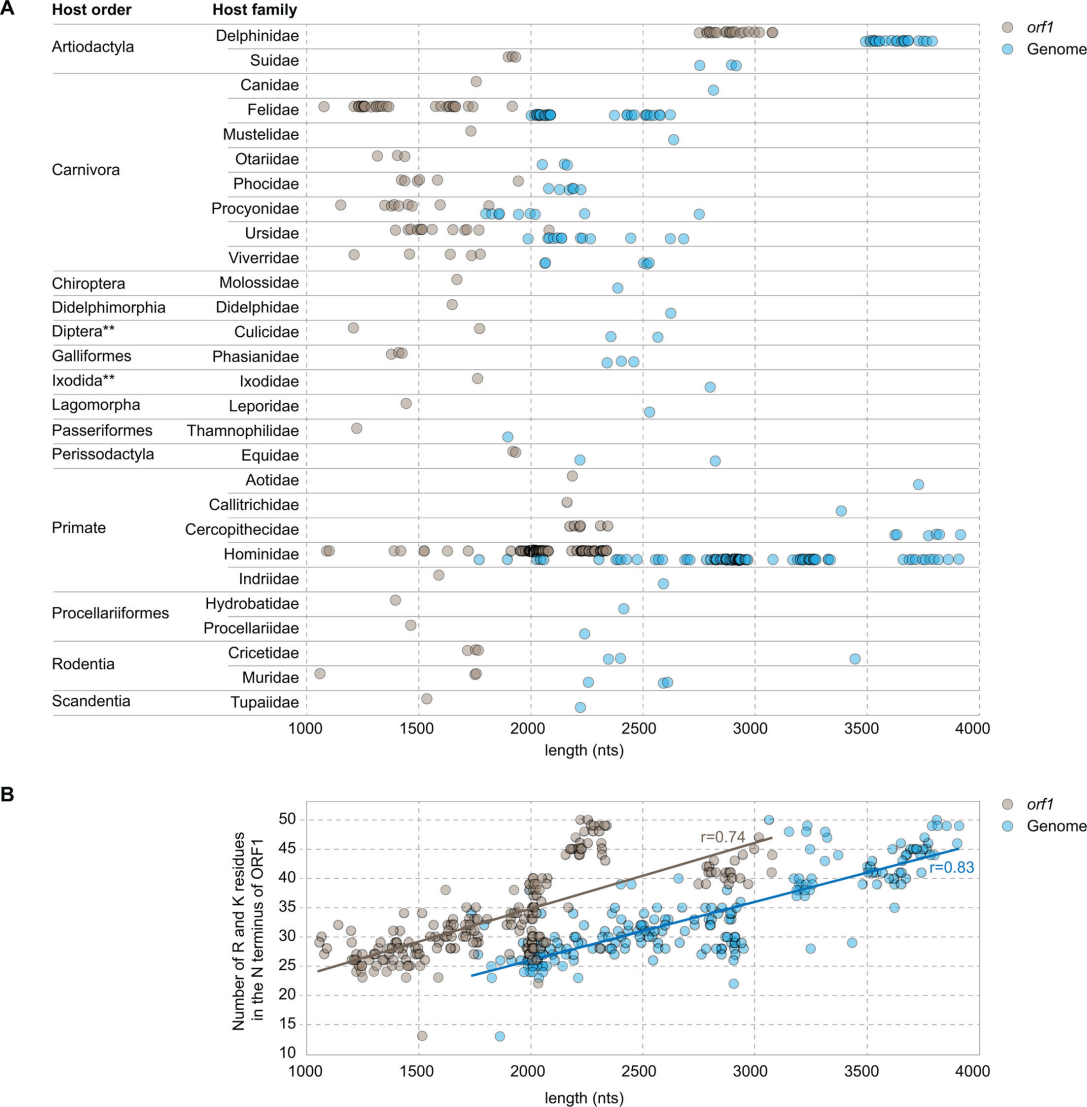
Anellovirus genome and *orf1* length distributions. (A) Distribution of anellovirus genome and *orf1* lengths is plotted for each host order and family. Circles represent individual anelloviruses that are representative of indicated host species. *The order Artiodactyla includes the Delphinidae family, which is associated with the 22 TTDelV species groupings of anelloviruses sequenced from oceanic dolphins in this study, and the Suidae family with three anelloviruses (GenBank accession numbers AB076001, AY823991, and JQ406846) identified in swine hosts, in separate studies. **The orders Diptera and Ixodida include invertebrate species, which are unlikely hosts for anelloviruses. Thus, such anelloviruses are likely derived from the blood meal of their vertebrate hosts. In this analysis, all Delphinidae family anelloviruses contain the largest ORF1-encoding genes. (B) Distribution of the number of arginine (R) and lysine (K) residues in the N-terminus of representative ORF1 proteins in relation to the *orf1* gene (*r* = 0.74 and *P*-value of 0.001 for the Pearson correlation) and genome (*r* = 0.83 and *P*-value of 0.001 for the Pearson correlation) sizes of anelloviruses (one from each species).

Comparative analysis of the ORF1 amino acid sequence lengths from representative species associated with 14 distinct host orders showed that those from Delphinidae anelloviruses have by far the largest ORF1 proteins detected to date. In TTDelVs, ORF1 proteins range in length from 910 to 1,020 amino acids (*orf1* genes 2,733–3,063 nts), whereas ORF1 from anelloviruses infecting all other hosts are considerably shorter (342–773 amino acids; *orf1* genes 1,028–2,022 nts; Fig. 4A). ORF1s of anelloviruses from Suidae family hosts (GenBank accession numbers AB076001, AY823991, and JQ406846), the closest mammalian relatives of cetaceans, contain 624–635 amino acids. Thus, ORF1s of Delphinidae-derived anelloviruses are 247 amino acids longer than the previously known largest ORF1 proteins (773 amino acids; *Alphatorquevirus cerco3*; KP296853) encoded by members of the *Alphatorquevirus* genus (3). Notably, Delphinidae-derived anelloviruses break the trend observed previously, namely, that the genome length of anelloviruses is correlated with the ORF1 length (3).

Analysis of the number of arginine (R) and lysine (K) residues in the N terminus of ORF1 relative to the genome and *orf1* gene lengths showed that there is a positive correlation (*r* = 0.83 for genome length and *r* = 0.74 for *orf1* gene length; Fig. 4B). This result is consistent with that obtained by Requião et al. (62) showing that the positively charged arginine domains located in the N-terminal region of capsid proteins of diverse RNA and DNA viruses are positively correlated with the genome size.

**Fig 5:**
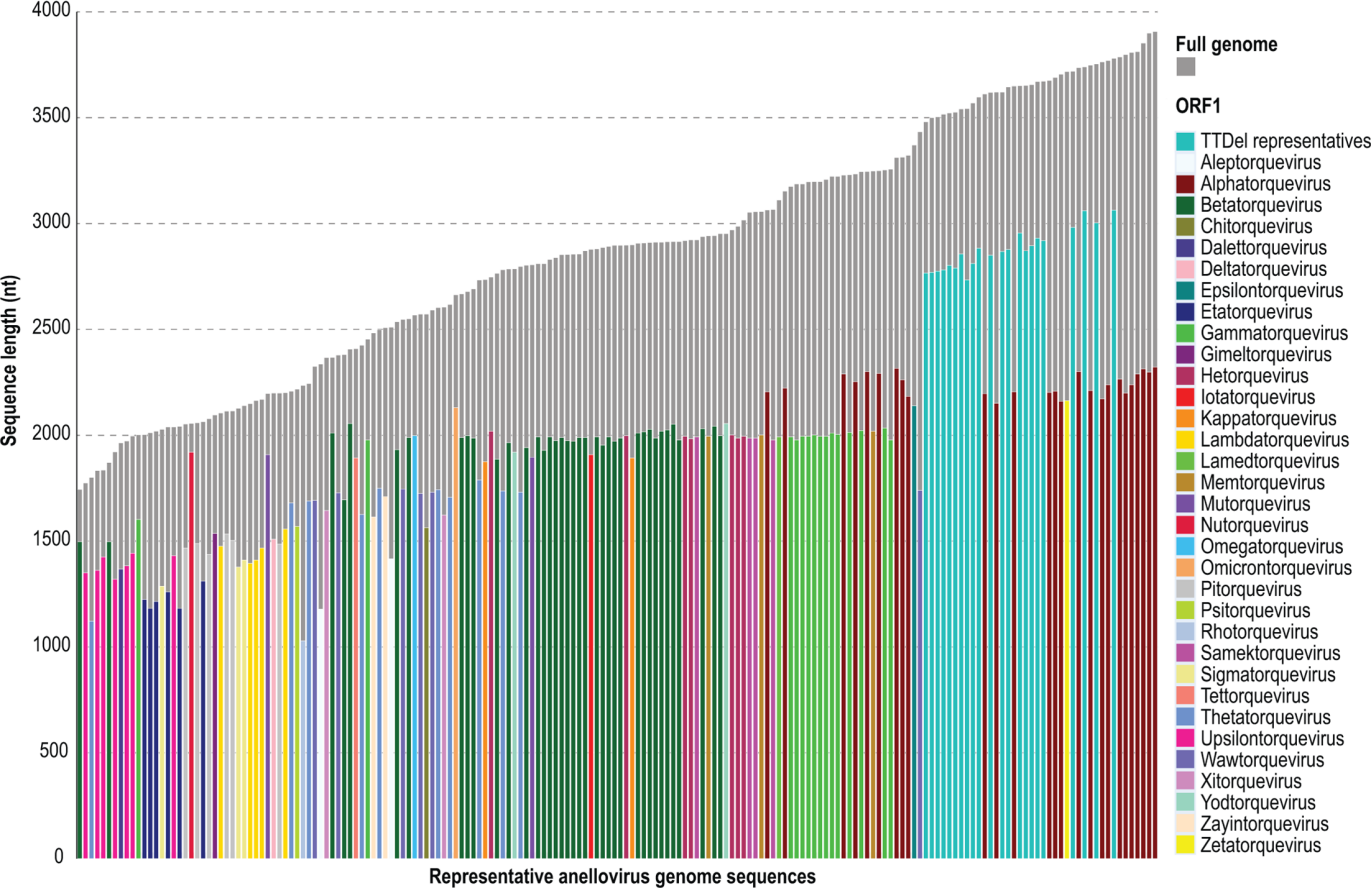
*orf1* to full genome length (nucleotide) ratio of representative anellovirus species. The 22 novel TTDel sequences (turquoise bars) were analyzed alongside 162 representative sequences from all 33 established anellovirus genera. *orf1* lengths and full genome lengths are organized by size.

Comparative analysis of the genomes and ORF1 lengths for representative species from each genus illustrates that although there is an overall positive relationship between genome and ORF1 lengths, there are a number of exceptions. For instance, the genomes of certain members of the *Alphatorquevirus*, *Epsilontorquevirus,* and *Zetatorquevirus* are larger than those of delphinid anelloviruses, yet their ORF1 proteins are substantially shorter. Overall, among the representative anellovirus sequences, *orf1* of TTDelV species is disproportionately larger than ORF1 of all previously described anelloviruses (turquoise bars; Fig. 5). In particular, the *orf1* in *Alphatorquevirus* members with similar sized or larger genomes are about 500 nts shorter than *orf1* sequences in TTDelV species (burgundy bars; Fig. 5). It does appear, however, that among Delphinid anelloviruses themselves, there is a positive correlation between *orf1* length and the genome length.

### ORF1 proteins of TTDelVs contain a conserved jelly-roll core and a hypervariable projection domain

**Fig 6:**
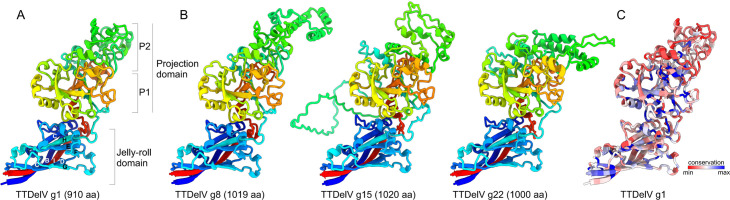
Structural models of selected TTDelV ORF1. (A) The central core of TTDelV ORF1 exemplified using the protein of TTDelV1, the smallest of the TTDelV ORF1s. The β-strands (B through I) constituting the jelly-roll domain are labeled. The jelly-roll and projection (P) domains as well as subdomains P1 and P2 are also indicated. (B) Structural models of the three largest ORF1 proteins encoded by TTDelVs. The sizes of the corresponding ORF1 proteins are indicated in parentheses. The structures in panels A and B are colored using the rainbow scheme from N-terminus (blue) to C-terminus (red). (C) Structural model of TTDelV1 ORF1 colored according to sequence conservation among TTDelVs representing the 22 species, with the more conserved and variable positions shown in blue and red, respectively.

To analyze the structural differences between the extravagantly large TTDelV ORF1 proteins and those encoded by other anelloviruses, we modeled the ORF1 structures of TTDelVs representing all 22 putative species described in this study using AlphaFold2 (see Materials and Methods). The predicted local distance difference test scores for the obtained models ranged between 94.9 and 96.6, indicating high confidence (Table S1). As observed in the previously analyzed anellovirus ORF1s (3), the ORF1 proteins of TTDelVs can be structurally dissected into the central core, consisting of the jelly-roll (JR) and projection (P) domains and extended N-terminal and C-terminal regions (Fig. 6A; Fig. S1). The canonical JR domain comprises eight antiparallel β-strands (B through I), which form two juxtaposed β-sheets, BIDG and CHEF (63, 64). In anelloviruses, the BIDG β-sheet is extended by an additional β-strand (C′) inserted between β-strands C and D, forming a five-stranded β-sheet C′BIDG. The P-domain is an insertion within the loop connecting the JR domain β-strands H and I (HI-loop). In the modeled monomer structures, the N- and C-terminal regions vary in terms of length, secondary structure elements, and their relative position with respect to the central core, suggesting flexibility in these regions, although within the virion, they likely occupy fixed positions. The same domain organization was found in all TTDelV ORF1 proteins, including the largest ones (>1,000 amino acids) encoded by TTDelV g8, TTDelV g15, and TTDelV g22 (Fig. 6B).

The JR domain plays a key role in the formation of the icosahedral T = 1 capsid, whereas the P domain points outward and is predicted to be important for host cell surface receptor recognition and/or evasion of host immune responses (3, 65). By contrast, the terminal regions are likely implicated in capsid assembly and stabilization through interactions with the neighboring subunits and genomic DNA. In particular, the N-terminal region is enriched in arginine residues and is likely to be implicated in genome binding and nuclear localization, as suggested for circoviruses (66). Below, we restricted our analyses to the central core, i.e., JR and P domains, of ORF1 (Fig. 6A).

Comparison of the TTDelV ORF1 homologs from different species revealed the conservation of the JR domain contrasted by extensive variability of the P domain, both in terms of topology and sequence conservation (Fig. 6; Data S2). The P domain can be further divided into two subdomains, P1 and P2. The P1 subdomain is proximal to the JR domain and serves as a platform for P2, which occupies the most distal position with respect to the virion shell (3, 65). While the P1 subdomain was largely conserved among TTDelV ORF1s, P2 displayed considerable variation, with distinct secondary structure elements predicted for different homologs, including additional α-helices, β-strands, and extended loops (Fig. S2). The P2 subdomain has been hypothesized to represent a point of contact with the host immune system (65) and is thus expected to be under constant pressure to change in order to avoid clearance. The observed hypervariance of P2 is consistent with this possibility.

### The larger sizes of TTDelV ORF1 are attributed to the expanded central core and C-terminal domains

**Fig 7:**
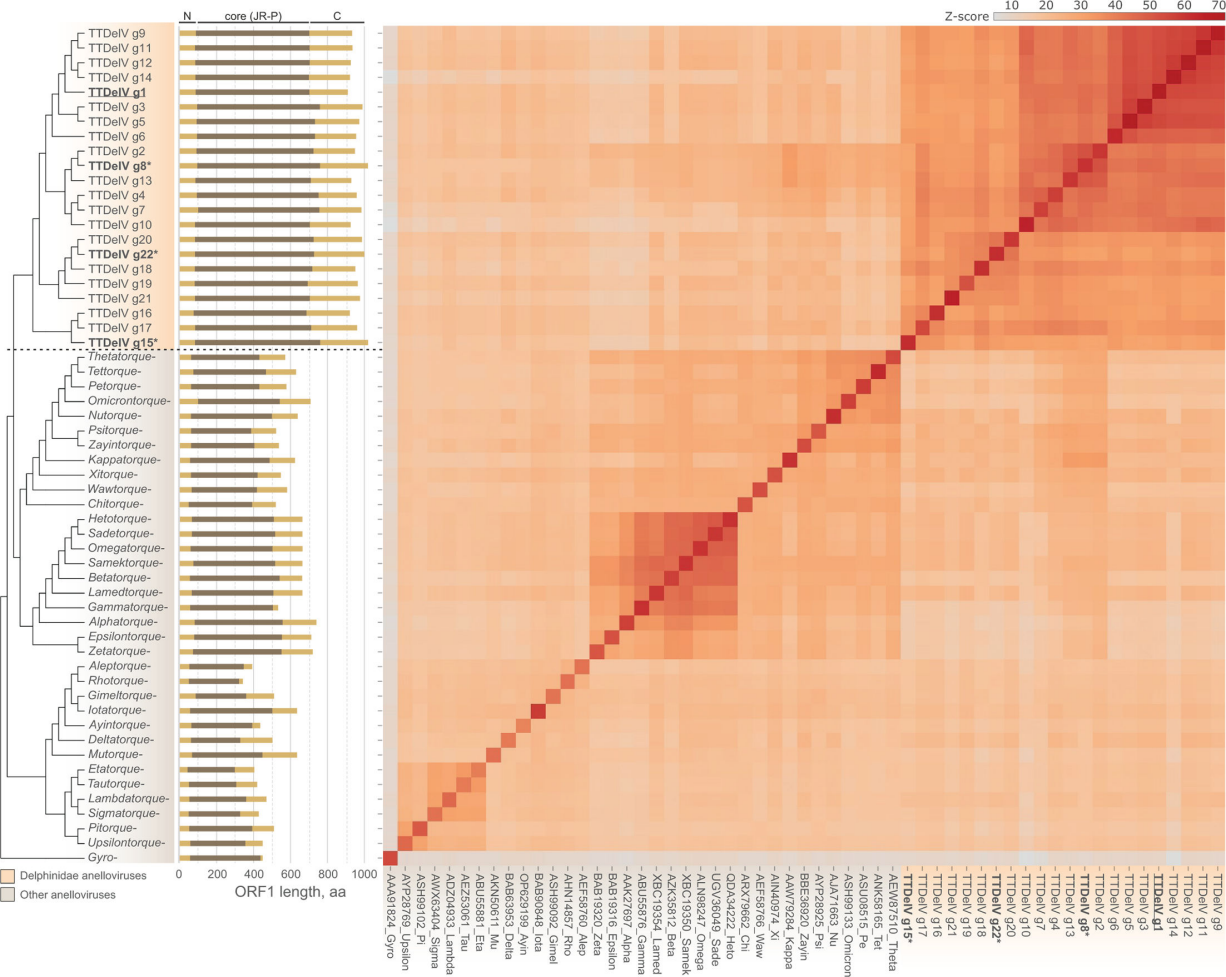
Structural relationships between ORF1 proteins of anelloviruses from different genera. Cluster dendrogram and heatmap were constructed on the basis of the comparison of pairwise *Z*-scores calculated using DALI. The previously classified anelloviruses and TTDelVs are indicated with different background colors on the dendrogram. The former are labeled with an abbreviated genus name, and the corresponding ORF1 GenBank accession numbers are provided at the bottom of the heatmap. The bar plot depicts the lengths of ORF1 proteins compared with the relative positions of the terminal (N and C) domains and the core region including the jelly-roll and projection (JR-P) domains indicated in light and dark brown, respectively. The three largest ORF1 homologs (>1,000 amino acids) are indicated with asterisks, whereas the smallest one (910 amino acids, TTDelV g1) is underlined.

**Fig 8:**
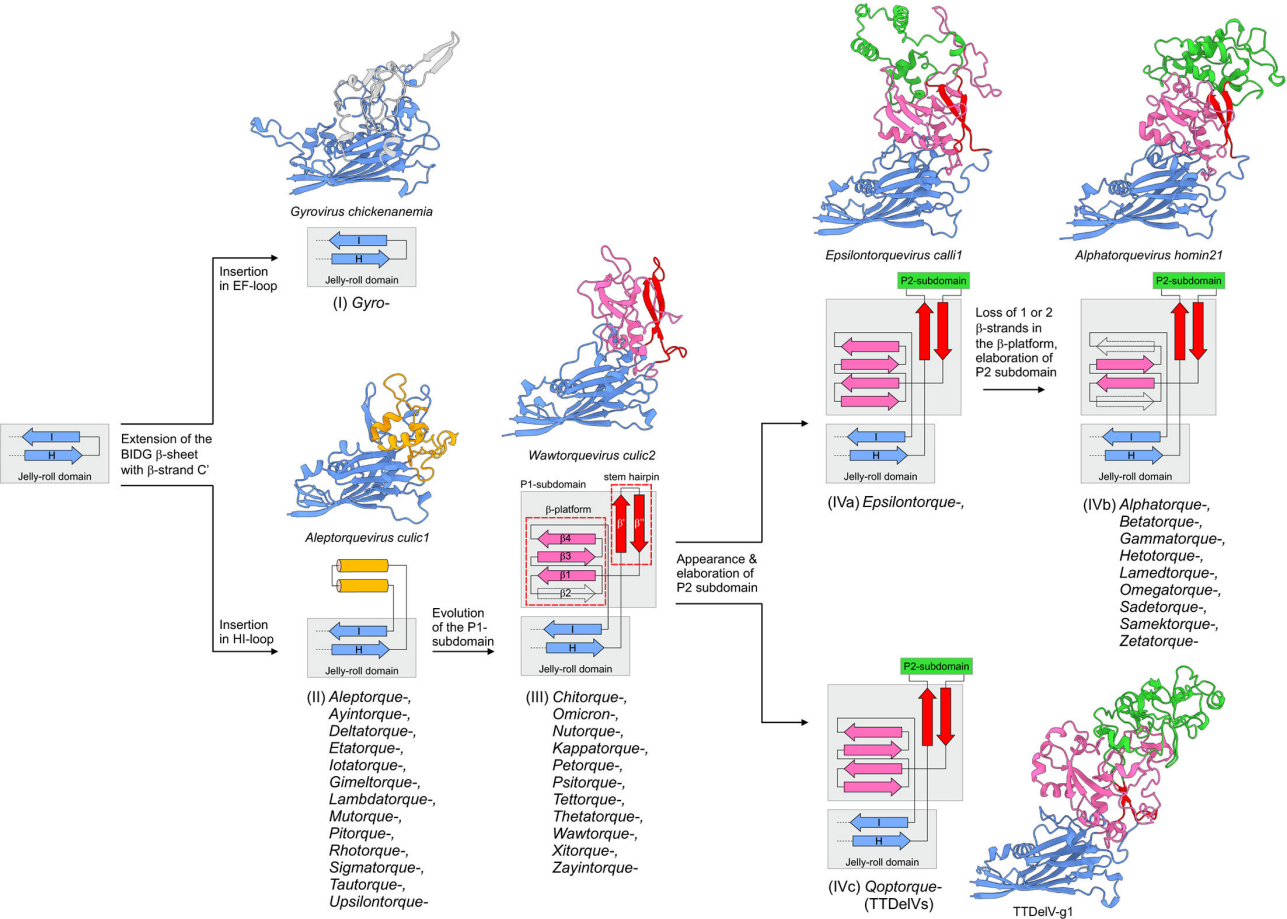
Putative evolutionary transitions in the capsid protein structure throughout anellovirus evolution. Each structural group is depicted by a structure of a representative ORF1 and a topological diagram of the insertion within the HI-loop of the jelly-roll domain. Only H and I β-strands of the jelly-roll domain are shown. Genera of anelloviruses that encode ORF1 of each structural group are listed below the corresponding topological diagram. The four major structural groups are indicated with Roman numbers, with group IV being further divided into three subgroups, IVa, IVb, and IVc. Major structural changes are explained next to the corresponding arrows. The structural models are colored according to the domain organization: jelly-roll domain, blue; β-platform and stem hairpin of the P1 subdomain, magenta and red, respectively; P2 subdomain, green; gyrovirus-specific insertion within the EF-loop, gray; α-helical insertion within the HI-loop, orange.

We next used DALI to compare the ORF1 structural models of TTDelVs to each other and the previously generated models of anellovirus capsid proteins representing all established genera (3). In this analysis, all TTDelV ORF1 homologs clustered together (Fig. 7), consistent with the sequence-based phylogenetic analysis (Fig. 2), further supporting the grouping of the 22 TTDelV species into a genus. Notably, the three largest TTDelV ORF1 homologs did not form a group, suggesting that their sizes increased independently of each other. Although the N-terminal regions were overall of similar lengths across all TTDelV ORF1 homologs, the central core and the C-terminal region varied in length. Comparison of the structural neighbors in the dendrogram showed that an increase in ORF1 size can occur by the expansion of either the central core (e.g., compare TTDelV g15 versus TTDelV g17 [679 versus 627 amino acids, respectively]) or the C-terminal region (e.g., compare TTDelV g13 and TTDelV g8 [223 versus 257 amino acids, respectively]) (Fig. 7). A similar pattern is also characteristic of anelloviruses from other genera. For instance, in the structure-based dendrogram and phylogeny, gammatorqueviruses consistently form a clade with alphatorqueviruses, betatorqueviruses, epsilontorqueviruses, hetotorqueviruses, zetatorqueviruses, and omegatorqueviruses (Fig. 2; Fig. 8). However, gammatorqueviruses encode ORF1 proteins that are >100 amino acids shorter than homologs from the other genera in this cluster, and the difference can be attributed to both smaller central core and C-terminal regions (Fig. 7).

The central core of TTDelV ORF1 is nearly twice as large as that of their homologs from other anelloviruses (on average 630 versus 361 aa, median 627 versus 348 amino acids) and is >100 amino acids larger than for the previous record holder, alphatorquevirus, with 500 amino acids. The C-terminal domains of TTDelV ORF1 are also considerably larger compared to anelloviruses from other genera, namely, on average 240 versus 124 amino acids (median 236 versus 134 amino acids). Although the N-terminal region of ORF1 in TTDelVs was slightly larger compared to other anelloviruses (on average 87 versus 65 amino acids), its contribution to the overall size of ORF1 is modest. Thus, the augmentation of the central core and the C-terminal domain is the key factor underlying the dramatic expansion in the size of TTDelVs ORF1.

### Analysis of the P-subdomains could guide higher rank classification of anelloviruses

We have previously shown that anelloviruses display dramatic variation in the complexity of the insertions within the HI-loop of the JR domains of ORF1 proteins and hypothesized that anelloviruses evolved from a circovirus-like ancestor with a simple JR capsid protein through acquisition and gradual augmentation of the P-domain (3). Comprehensive comparison of the predicted ORF1 structures suggests that anelloviruses can be broadly divided into four groups: (i) viruses with an insertion into the EF-loop that lack insertion into the HI-loop, represented by members of the *Gyrovirus* genus; (ii) viruses with a simple α-helical insertion into the HI-loop of the JR domain; (iii) viruses with a simple P domain consisting of the P1-subdomain alone; and (iv) viruses with an elaborate P-domain containing both P1 and P2 subdomains (Fig. 8). The latter group can be further subdivided into three subgroups based on the complexity of the P2 subdomain, and the evolution of the more complex P-domain from the simpler state through gradual augmentation can be plausibly reconstructed.

The P1 subdomain consists of a conserved core of three- or four-stranded β-sheet and a β-hairpin (stem hairpin), with the two elements being roughly perpendicular to each other. The P2 subdomain is an insertion into the apex of the loop in the P1 stem hairpin, with the β-sheet serving as a platform onto which the P2 subdomain is mounted (Fig. 8). Viruses from the genera *Wawtorqueviruses*, *Zayintorquevirus*, and *Psitorquevirus* might resemble the ancestral state of the P1 subdomain, with the stem hairpin lacking any pronounced insertions in the loop. By contrast, in a number of anellovirus genera, the P1 subdomain consists of a four-stranded β-sheet and the loop connecting the two β-strands of the stem hairpin is more extended, although still lacking extensive secondary structure other than random coil. By contrast, epsilontorqueviruses already contain a more elaborate P2 subdomain with several α-helices. In phylogenetic analyses (Fig. 2), epsilontorqueviruses together with zetatorqueviruses are basal to a clade including alphatorqueviruses, betatorqueviruses, gammatorqueviruses, hetotorqueviruses, omegatorqueviruses, sadetorqueviruses, samektorqueviruses, and lamedtorqueviruses. Notably, with the exception of epsilontorqueviruses, ORF1s of all these anelloviruses, including zetatorqueviruses, have apparently lost one or two β-strands of the four-stranded β-sheet of the P1 subdomain. The TTDelV ORF1 proteins display an organization of the P1 subdomain most similar to that of epsilontorqueviruses, with a four-stranded β-sheet in the P1 subdomain. However, both in phylogenetic analyses and in the structural clustering dendrogram, TTDelVs consistently cluster outside of the group IV anelloviruses with the large ORF1s (Fig. 2 and 7). Thus, it appears that TTDelVs did not evolve directly from epsilontorqueviruses or other viruses in this group but rather convergently augmented their P2 subdomains.

Due to the high sequence divergence, including extensive size variation, of ORF1 proteins, reconstruction of deeper evolutionary relationships between viruses from different genera of *Anelloviridae* proves challenging, with phylogenetic analyses systematically failing to resolve the deep branching points. Detailed structural comparisons can aid higher rank classification of anelloviruses. For instance, the four structural groups of anelloviruses presented above potentially could be unified into separate subfamilies.

### Conclusion

In this study, we characterized a novel group of Delphinidae-infecting anelloviruses. According to ORF1 pairwise nucleotide sequence comparisons, the amino acid sequence phylogeny, and comparisons of the ORF1 structural models, the 69 recovered Delphinid viral genomes were categorized into 22 species-level assemblages (TTDelV g1−22). In the phylogenetic trees, the TTDelV species groupings cluster together, forming a putative new genus, which we propose naming “*Qoptorquevirus*.” The TTDelV species groupings correspond to the cetacean host species, suggesting virus-host coevolution. Furthermore, we identified diverse co-infections within the infraorder Cetacea, which aligns with anellovirus infection patterns found in other vertebrates.

Remarkably, the Delphinid anelloviruses encode ORF1 proteins that are much larger than those of other known anelloviruses, including primate anelloviruses with similar genome sizes. Analysis of the structural models of the Delphinid anelloviruses showed that they have the same characteristic ORF1 domains as other anelloviruses, namely, the central core, the extended N-terminal positively charged region, and the extended C-terminal region. The central core consists of the conserved JR domain and the variable P domain. The JR-domain plays a key role in capsid assembly and is therefore subject to strong purifying selection. In contrast, the P-domain likely interacts with the host immune system and/or cell surface receptors and likely evolves under diversifying selective pressure to evade the immune response. Consistent with this notion, we observed hypervariability in the P-domain of the Delphinid anelloviruses. Our observations suggest that Delphinid anelloviruses evolved the large, elaborate P-domains convergently with the primate anelloviruses. Finally, we argue that structural comparisons of the ORF1 proteins can guide higher-level classification of anelloviruses, which proved to be challenging based on the nucleotide or amino acid sequence comparisons due to their extreme divergence. To conclude, here we expand the identified host range of anelloviruses to Delphinidae family members and provide insight into their virus-host interactions, evolution, and classification.

## Data Availability

Anellovirus genomes have been deposited in GenBank under accession numbers PP790224-PP790292. The raw reads have been deposited under BioProject PRJNA1189461, BioSample numbers SAMN44970975, SAMN44970976, SAMN4497077, and SAMN44970978, and SRA numbers SRR31500901, SRR31500900, SRR31500899, and SRR31500898.

## SUPPLEMENTAL MATERIAL

Data Set S1 jvi.01370-24-s0001.xlsx ORF1 pairwise identity matrix.

Data Set S2 jvi.01370-24-s0002.xlsx Recombination breakpoints.

Figure S1 jvi.01370-24-s0003.pdf Structural model of the TTDelV1 ORF1.

Figure S2 jvi.01370-24-s0004.pdf Structural diversity of the P-domains from TTDelVs representing all 22 species.

Table S1 jvi.01370-24-s0005.pdf pLDDT scores.

ASM does not own the copyrights to Supplemental Material that may be linked to, or accessed through, an article. The authors have granted ASM a non-exclusive, world-wide license to publish the Supplemental Material files. Please contact the corresponding author directly for reuse.
