# Supplementary material for: Novel lineage of anelloviruses with large genomes identified in dolphins
Source: J Virol. 2024 Dec 12;99(1):e01370-24. doi: 10.1128/jvi.01370-24 (PMC11784456; doi:10.1128/jvi.01370-24)
Supplement: Figure S1 — Structural model of the TTDelV1 ORF1. [file jvi.01370-24-s0003.pdf]

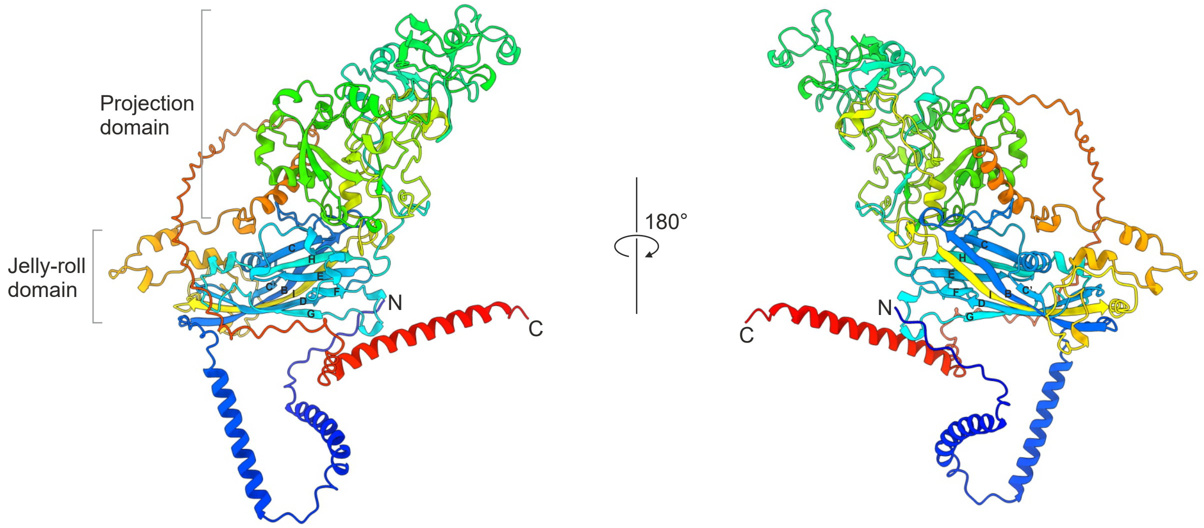

Supplementary Fig 1. Structural model of the TTDeIV1 ORF1. The structure is colored using the rainbow scheme from N-terminus (blue) to C-terminus (red). The β-strands (B through I) constituting the jelly-roll domain are labeled. The jelly-roll and projection domains are also indicated.
