# Supplementary material for: Novel lineage of anelloviruses with large genomes identified in dolphins
Source: J Virol. 2024 Dec 12;99(1):e01370-24. doi: 10.1128/jvi.01370-24 (PMC11784456; doi:10.1128/jvi.01370-24)
Supplement: Figure S2 — Structural diversity of the P-domains from TTDelVs representing all 22 species. [file jvi.01370-24-s0004.pdf]

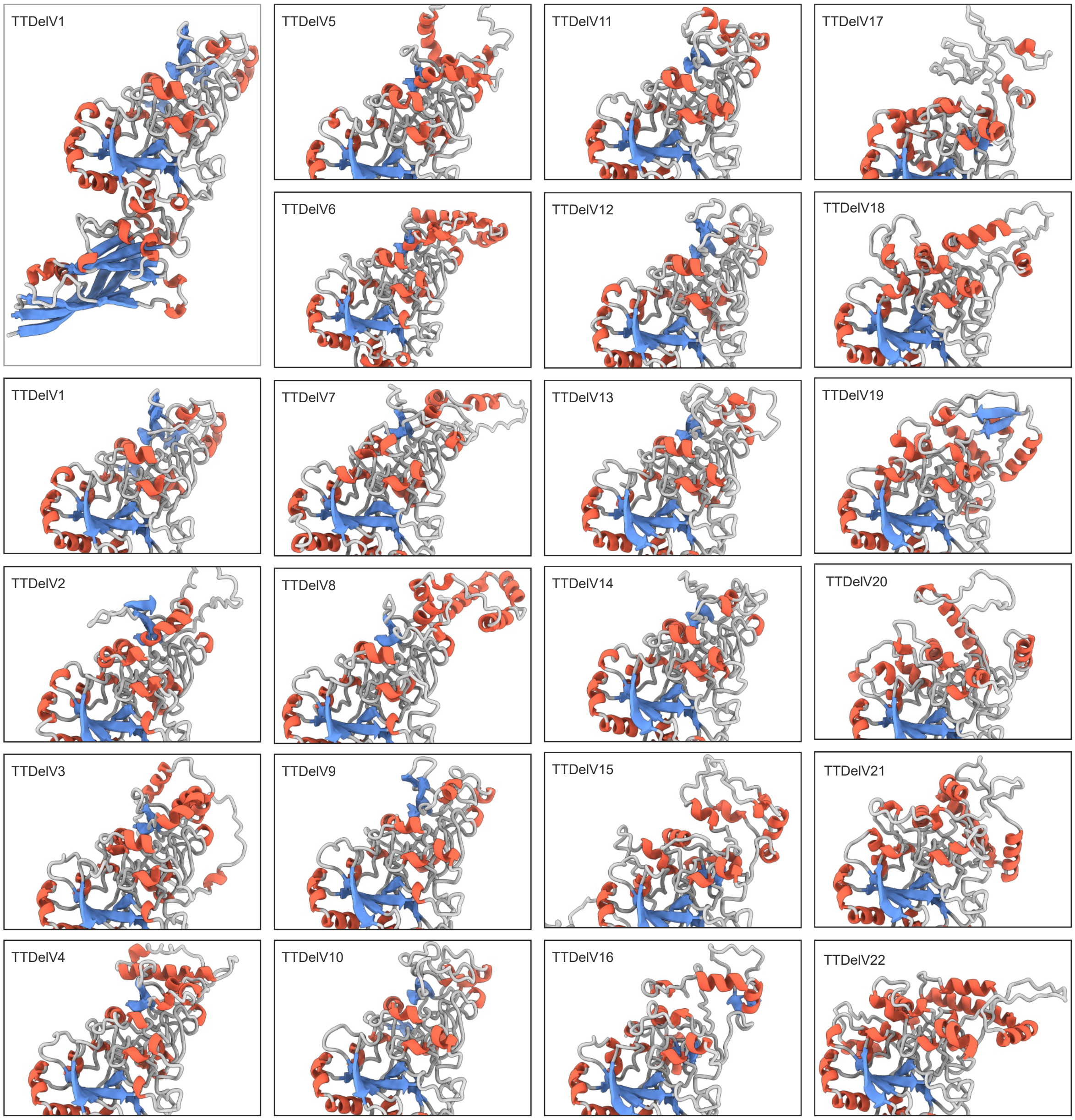

Supplementary Fig 2. Structural diversity of the P-domains from TTDelVs representing all 22 species. In the top left, the full structure of ORF1 from TTDelV1 is shown for reference. All ORF1 proteins were superposed according to their jelly-roll domains and thus all depicted P-domains have the same relative orientation. The structural models are colored according to the secondary structure elements: α-helices, red; β-strands, blue; random coil, grey.
