# Supplementary material for: Novel lineage of anelloviruses with large genomes identified in dolphins
Source: J Virol. 2024 Dec 12;99(1):e01370-24. doi: 10.1128/jvi.01370-24 (PMC11784456; doi:10.1128/jvi.01370-24)
Supplement: Table S1 — pLDDT scores. [file jvi.01370-24-s0005.pdf]

Supplementary Table 1. pLDDT scores. Summary of the pLDDT scores for the obtained models.

	pLDDT
TTDelV g1	pLDDT=96.4
TTDelV g2	pLDDT=96.3
TTDelV g3	pLDDT=95.6
TTDelV g4	pLDDT=95.4
TTDelV g5	pLDDT=95.4
TTDelV g6	pLDDT=96.6
TTDelV g7	pLDDT=95.9
TTDelV g8	pLDDT=95.8
TTDelV g9	pLDDT=96.1
TTDelV g10	pLDDT=96.1
TTDelV g11	pLDDT=96
TTDelV g12	pLDDT=96.5
TTDelV g13	pLDDT=96.2
TTDelV g14	pLDDT=96.4
TTDelV g15	pLDDT=94.9
TTDelV g16	pLDDT=95.8
TTDelV g17	pLDDT=95
TTDelV g18	pLDDT=95.5
TTDelV g19	pLDDT=95.7
TTDelV g20	pLDDT=95.9
TTDelV g21	pLDDT=96
TTDelV g22	pLDDT=96
